# Isotopic composition of serum zinc and copper in healthy children and children with autism spectrum disorder in North America

**DOI:** 10.3389/fnmol.2023.1133218

**Published:** 2023-02-15

**Authors:** Kerri Miller, Patrick L. Day, Supriya Behl, Lindsay Stromback, Adriana Delgado, Paul J. Jannetto, Michael E. Wieser, Sunil Q. Mehta, Mukesh K. Pandey

**Affiliations:** ^1^Isotope Science Laboratory, Department of Physics and Astronomy, University of Calgary, Calgary, AB, Canada; ^2^Metals Laboratory, Department of Laboratory Medicine and Pathology, Mayo Clinic, Rochester, MN, United States; ^3^Children’s Research Center, Department of Pediatric and Adolescent Medicine, Mayo Clinic, Rochester, MN, United States; ^4^Division of Developmental and Behavioral Pediatric, Department of Pediatrics and Adolescent Medicine, Mayo Clinic, Rochester, MN, United States; ^5^Department of Psychiatry and Psychology, Mayo Clinic, Rochester, MN, United States; ^6^Division of Nuclear Medicine, Department of Radiology, Mayo Clinic, Rochester, MN, United States

**Keywords:** isotopic composition, isotope fractionation, zinc, copper, autism spectrum disorder

## Abstract

To better understand zinc and copper regulation and their involvement in various biochemical pathways as it relates to autism spectrum disorder (ASD), isotopic composition of serum zinc and copper were evaluated in both healthy children and children with ASD in North America. No significant difference in isotopic composition of serum zinc or copper with respect to healthy controls and ASD children were identified. However, the isotopic composition of serum copper in boys was found to be enriched in ^65^Cu in comparison to previously published healthy adult copper isotopic composition. Furthermore, in both boys and girls, the average isotopic composition of serum zinc is heavier than previously published healthy adult isotopic zinc composition. There was also a negative association between total zinc concentrations in serum and the zinc isotopic composition of serum in boys. Finally, children with heavier isotopic composition of copper also showed a high degree of variability in their zinc isotopic composition. While numerous studies have measured the isotopic composition of serum zinc and copper in adults, this is one of the first studies which measured the isotopic composition of serum copper and zinc in children, specifically those diagnosed with ASD. The results of this study showed that age and gender specific normal ranges of isotopic composition must be established to effectively use isotopic composition analysis in studying various diseases including ASD.

## Introduction

In the United States, the prevalence of autism spectrum disorder (ASD) is 1.9% and has increased since 2000 (Maenner et al., 2020). Not only are there significant clinical conditions experienced by ASD patients including intellectual disability, language problems, and increased risk of epilepsy and GI disorders, including constipation, diarrhea, and abdominal pain, but ASD also presents a significant economic burden for the individual patient, the patient’s family and society (Wasilewska and Klukowski, 2015). It is estimated that the annual direct medical, direct non-medical and productivity costs associated with ASD will be 461 billion for the year 2025 (Leigh and Du, 2015). It is thus imperative to extensively study the etiology and progression of ASD to improve the timely diagnosis and treatment of these patients. While there is a strong genetic influence seen for ASD, there also has been a growing body of research dedicated to trace metals (including Cu, Zn, Fe and Se) and their association with the incidence of ASD (Behl et al., 2020).

Zinc for example is an essential trace element that is involved in glutamatergic transmission during embryonic and childhood development and has been extensively studied as it relates to ASD (Behl et al., 2020). For example, in a study of 1,967 children with ASD, nearly 30% of children with ASD had low concentrations of zinc present in hair samples (Yasuda et al., 2011). However, a study previously conducted by our group that involved 64 children with ASD and 65 non-ASD controls, did not find any significant difference between serum or hair zinc levels in children with ASD compared to age-matched and sex-matched controls (Mehta et al., 2021). Furthermore, one study, including 27 children with ASD and 27 non-ASD control, found higher levels of zinc in the hair of ASD patients when compared to controls (Al-Farsi et al., 2013). These differences may be the result of study methodology differences or by geographic-specific factors including differences in nutrition, economic status, and associated illnesses (Behl et al., 2020).

Copper is another essential trace element that is necessary for enzymes to control processes such as respiration, antioxidant defense, and neuropeptide processing (Puchkova et al., 2018). Like zinc, it also has been extensively studied in respect to ASD. For example, a study of 230 children with ASD, pervasive developmental disorder and Asperger’s syndrome found that 15.4% of children with ASD had higher copper plasma levels than the reference range (Faber et al., 2009). A study by Li et al., that included 60 patients with ASD and 60 age and sex-matched controls, also showed that children with ASD had elevated copper serum levels when compared to healthy sex matched and age matched controls (Li et al., 2014). However, a study by our group, that included 64 children with ASD and 65 non-ASD controls, did not find significant differences in copper concentrations in serum or hair of North American children with ASD when compared to healthy controls (Mehta et al., 2021).

Considering multiple ASD studies with conflicting copper and zinc data, new analytical techniques must be employed to further study the potential role of copper zinc or other trace elements, like iron, may have on the etiology and progression of ASD. One emerging technique in the field of biologic trace element research that may aid in the study of trace elements in ASD patients is the measurement of stable isotope composition by multicollector inductively coupled plasma mass spectrometry (MC-ICP-MS). The incorporation of isotopic composition in the study of the progression of ASD can provide important insights into the metabolic processes responsible for the trafficking of metals in the body. Isotopes of an element are atoms with the same number of protons but different numbers of neutrons. Isotopes behave the same chemically because they have identical electronic configurations. However, the mass difference due to the different numbers of neutrons, can result in a redistribution of the isotopes due to mass dependent effects in biochemical reactions (Albarède et al., 2017). Copper has two stable isotopes (^63^Cu and ^65^Cu), and zinc has 5 stable isotopes (^64^Zn, ^66^Zn, ^67^Zn, ^68^Zn, and ^70^Zn). In the context of a biological system, this results in a redistribution of the isotopes among different proteins, cells, and ultimately organs. The redistribution of atoms is known as isotopic fractionation and the extent of isotope abundance variation is typically represented as a delta value, which is the relative difference between the isotopic composition of the sample and a standard reference material. For example, in the case of copper, the delta value is defined as:


(1) $$\delta C65u_{AE633}=\left(\frac{R_{Sample}}{R_{AE633}}-1\right)*1000$$

where *R_Sample_* and *R_AE_*_633_ are the isotope amount ratios of ^65^Cu to ^63^Cu for the sample and reference materials, respectively. The delta value reflects the subtle, but significant variations in the isotope amount ratios and the relative differences are expressed in parts per thousand (or per mil, ‰). The isotopic compositions that are measured are those of atoms naturally present in the living system and no radioactive or enriched tracers are added.

Copper and zinc are essential metals that may be toxic at elevated concentrations. The processing of these metals, especially copper, is tightly controlled to ensure that the metals are not unbound within cells where they could cause damage through oxidative stress (Uriu-Adams and Keen, 2005). In a healthy system, the regulation of copper and zinc results in the observed isotopic compositions in specific compartments (e.g., serum) to fall within a small range. However, if there is a change in the bonding environment of the element or change in the protein pathway involving the metal, there may be a significant change in isotopic composition. Theoretically the extent of isotopic fractionation due to different binding motifs (for example, different metal-binding sites of proteins) have been characterized using computation methods (Fujii et al., 2013; Tennant et al., 2017). Experimentally, evidence for systematic changes in isotopic fractionation due to disruptions or malfunctions in the biological processing of the metal within the system have been documented for cell cultures, animal models, and in humans. Yeast cells have been used to demonstrate that the primary mechanism that results in copper isotope fractionation during import into cells is the action of copper transporters. The extent of the isotope fractionation is dependent on the ability of the cell to control the oxidation of the copper *via* reductase proteins (Cadiou et al., 2017). In mice, an antibiotic treatment was shown to modulate the expression levels of copper transporter 1 (CTR-1) in intestinal epithelial cells, which induced a large isotope fractionation compared to wild type mice (Miller et al., 2019). In humans, analyses have been done on the serum of adult patients and differences in serum copper isotopic composition have been observed in several types of cancers, Wilson’s disease, amyotrophic lateral sclerosis, liver cirrhosis, non-alcoholic liver fatty liver disease, and for those females with increased risk of preterm birth (Balter et al., 2015; Télouk et al., 2015; Bondanese et al., 2016; Sauzéat et al., 2018; Lamboux et al., 2020; García-Poyo et al., 2021; Kazi Tani et al., 2021; Wang et al., 2022).

The extent of isotope fractionation observed for zinc is smaller than that observed for copper isotopes. Unlike copper, zinc exists in only a single oxidation state and changes in oxidation state often can cause increase isotope composition (Büchl et al., 2008). Nevertheless, changes in zinc isotopic composition have been connected to disease. For example, in a study measuring the urine from 22 prostate cancer patients, 16 breast cancer patients, 14 benign breast disease patients and compared to age matched controls, the Zn isotopic composition of urine has shown a characteristic shift in those patients with pancreatic and prostate cancers (Schilling et al., 2021). It is hypothesized that the increased oxidative stress within cancer cells causes oxidation of metalloproteins with sulfur-rich ligands, which are relatively enriched in lighter zinc isotope. These lighter zinc atoms are released into the bloodstream and subsequently excreted in urine, resulting in *δ*^66^Zn values that are much lower than healthy individuals (Schilling et al., 2021). Patients in a study with breast cancer did not show a similar trend, suggesting that these cells were better equipped to deal with oxidative stress. Biopsies of 10 patients with Alzheimer’s brain tissue from persons with Alzheimer’s disease have shown copper and zinc isotopic fractionations that reflect the Braak stage of neurodegeneration with a shift towards lower *δ*^65^Cu values and higher *δ*^66^Zn values as the disease progressed (Moynier et al., 2020).

In the present study, we evaluated the isotopic composition of zinc and copper metal ions in both healthy children (*n* = 10) and children diagnosed with ASD (*n* = 10) to investigate their involvement either directly or indirectly in the pathophysiology of ASD. Present findings may provide additional insight into the role of biometals and their isotopic composition in ASD.

## Materials and methods

### Patient recruitment and selection

As was detailed in our previous study, children aged 24–48 months and newly diagnosed with ASD were recruited from the Dana Neurodevelopmental Disorders program at Mayo Clinic (Mehta et al., 2021). Age-matched controls were also recruited at an outpatient community pediatric clinic. From this original study population (64 patients with a diagnosis of ASD and 65 control patients), 5 male patients with ASD, 5 male control patients, 5 female ASD patients, and 5 female control serum samples were randomly selected for this follow-up study. All samples for this study were collected through informed consent approved by the Mayo Clinic Institutional Review Board (17-002689).

### Sample collection

Human serum samples were collected using a BD royal blue top clot activator vacutainer tube (Franklin Lakes, NJ). Serum from these tubes was allowed to clot, was centrifuged and then poured off into a metal-free specimen vial and stored frozen until analysis. The analysis of total copper and zinc in serum was conducted in an ISO class 7 cleanroom in order to reduce the risk of potential metal contamination.

### Analytical methods and chemicals

#### Total copper and zinc serum analysis by inductively coupled plasma mass spectrometry

A validated and clinically available laboratory developed test using Inductively Coupled Plasma Mass Spectrometry (ICP-MS) was used to measure copper and zinc in bulk serum. A PerkinElmer Elan or PerkinElmer NexION 350D ICP-MS spectrometer (Waltham MA) was used for all analyses. This assay used six calibration standards to establish an analytical measurement range of 0–5,000 ng/ml for both copper and zinc, respectively. Utak Trace Elements Serum Toxicology controls (Valencia, CA) were analyzed with each calibration and sample run to monitor the accuracy of the method. This assay was developed, and the performance characteristics were established in a manner to meet Clinical Laboratory Improvement Amendments (CLIA) requirements. A detailed description of the ICP-MS method used for the quantification of total copper and zinc in the serum of these samples has been described in a previous publication by our group (Mehta et al., 2021).

#### Copper and zinc isotope measurement of serum by MC-ICP-MS

Preparation of the serum samples for isotopic analysis were performed in a clean room using high-purity nitric acid prepared by double-subboiling reagent grade nitric acid (VWR, United States) in a Savillex acid purification system. High-purity hydrochloric acid and water were purchased from BDH and VWR. All plastics and quartz were leached with 6 M HCl for minimum 1 week prior to use to minimize zinc and copper blanks. Serum samples were digested in a CEM SP-D Discover microwave digestion system using 2 ml of concentrated nitric acid to ensure complete digestion of the serum proteins. Digestion was ramped to 200°C for 3 min and held at this temperature for 4 min.

Copper analysis: An aliquot of the digested serum containing 100 ng of Cu was ion-exchanged using a slightly modified version of the procedure outlined in (Miller et al., 2016). Briefly, 250 ul of Cu-specific resin (Triskem International, France) was used for the isolation of Cu from the matrix. Resin was precleaned with 4 ml of 6 M HCl before samples were loaded in 0.005 M HCl, matrix was washed with 10 ml of 0.005 M HCl, and copper was eluted with 2.5 ml of 6 M HCl.

Zinc analysis: An aliquot of the digested serum containing 300 ng of Zn was spiked with the appropriate amount of a Zn double spike (^64^Zn and ^67^Zn) to achieve a sample-to-spike ratio of 50% (± 5%). The mixture was equilibrated for a minimum of 5 h and then evaporated to dryness. Isolation of Zn for isotopic analysis was done by ion exchange chromatography using glass micro-columns packed with 250 μl of pre-cleaned AG MP 1 (100–200 mesh size, Bio-Rid, Hercules, United States). The ion exchange procedure was modified after a similar method by Moore et al. (2017).

Analysis of Cu and Zn isotopic composition were performed on a Neptune MC-ICP-MS (Thermo-Fisher, Bremen, Germany) using a Teflon APEX desolvating nebulizer (ESI, Omaha, USA). Instrumental mass bias for Cu was corrected using the sample-standard bracketing technique with ERM-AE633 as the bracketing standard and the *δ*^65^Cu values calculated as shown in Equation 1. Mass bias for Zn was corrected by inverting the double spike equations using in-house developed software (Rudge et al., 2009). Zinc isotopic composition is expressed as a *δ*^66^Zn value and calculated as follows:


(2) $$\delta Z66n_{IRMM3702}=\left(\left({\left(\frac{m_{66Zn}}{m_{64Zn}}\right)}^{\left(\alpha_{Sam}-\alpha_{IRMM3702}\right)}\right)-1\right)*1000$$

where *m* are the atomic masses of the two isotopes, and $\alpha_{Sam}$ and $\alpha_{IRMM3702}$ are the calculated fractionation factors for the sample and standard found from the inversion of the double spike equations.

Quality Control and uncertainty in isotopic measurements: The standard reference materials ERM-AE633 and ERM-AE647 (both purchased from Sigma-Aldrich, US) were used for Cu and IRMM 3702 (IRMM, Belgium) for Zn. For Cu, the processed standards had mean *δ*^65^Cu values (*n* = 4) of 0.01‰ ± 07 and 0.22‰ ± 02 for ERM-AE633 and ERM-AE647, respectively. Copper blanks were consistently below 1 ng for the analytical procedure. For Zn, processed IRMM-3702 had an average measured *δ*^66^Zn value of 0.03‰ (*n* = 3). Blanks were below 2 ng Zn for the analytical procedure. For both copper and zinc, the propagation of analytical uncertainty resulted in a standard uncertainty u(*δ*^65^Cu, *δ*^66^Zn) = 0.1‰ with a coverage factor of 2.

### Statistical analysis

Demographic continuous variables are summarized and reported as median along with the first and third quartiles. Total copper and zinc serum concentrations as well as *δ*^65^Cu and *δ*^66^Zn values are summarized by the mean and standard deviation (SD). Categorical variables were summarized as counts and percentages. ASD and control groups were compared utilizing Wilcoxon rank sum tests for continuous variables and Fisher’s exact tests for categorical. Kendall Tau-b correlations were used to evaluate total element concentration correlation with isotopic composition values. For all hypothesis testing, a nominal *p* < 0.05 was considered statistically significant. All analyses were performed using SAS statistical software (version 9.4; SAS Institute Inc., Cary, NC) and graphics were created using R v3.6.2 statistical software (R Core Team, Vienna Austria).

## Results

The study cohort included 10 children with ASD and 10 age and gender-matched controls (Table 1). Ages ranged from 25 to 46 months with a median of 40. Majority of patients self-identified as White race (16/20) and not Hispanic or Latino (19/20). BMI at age of sample collection ranged from 13.9 to 18.7 (median 16.4). Median height at age of sample collection was 97.4 cm and median weight was 15.1 kg. Within each gender, age, race, ethnicity, and BMI were not significantly different for ASD patients compared to control patients (each *p* ≥ 0.12).

**Table 1:** Characteristics of case and control subjects.

	Total (*N* = 20)	ASD boys (*N* = 5)	Control boys (*N* = 5)	*p*-value	ASD girls (*N* = 5)	Control girls (*N* = 5)	*p*-value
Race				0.44^1^			>0.99^1^
African American	1 (5.0%)	0 (0.0%)	0 (0.0%)		1 (20.0%)	0 (0.0%)	
Asian Other	1 (5.0%)	1 (20.0%)	0 (0.0%)		0 (0.0%)	0 (0.0%)	
Asian Thai	1 (5.0%)	1 (20.0%)	0 (0.0%)		0 (0.0%)	0 (0.0%)	
Other	1 (5.0%)	0 (0.0%)	0 (0.0%)		0 (0.0%)	1 (20.0%)	
White	16 (80.0%)	3 (60.0%)	5 (100.0%)		4 (80.0%)	4 (80.0%)	
Ethnicity				--			>0.99^1^
Mexican	1 (5.0%)	0 (0.0%)	0 (0.0%)		0 (0.0%)	1 (20.0%)	
Not Hispanic or Latino	19 (95.0%)	5 (100.0%)	5 (100.0%)		5 (100.0%)	4 (80.0%)	
Weight (kg)				0.39^2^			0.020^2^
Median	15.1	13.4	16.3		16.5	14.3	
Q1, Q3	13.6, 17.4	12.8, 16.5	14.4, 17.6		16.1, 19.4	13.2, 14.7	
BMI (kg/m^2^)				0.77^2^			0.71^2^
Median	16.4	16.8	16.4		16.3	15.8	
Q1, Q3	15.8, 16.9	15.9, 18.1	15.7, 17.1		16.2, 16.9	14.6, 16.9	
Height (cm)				0.60^2^			0.11^2^
Median	97.4	92.3	97.9		101.0	95.3	
Q1, Q3	92.3, 102.3	88.5, 97.2	89.3, 103.0		100.9, 107.1	89.4, 99.3	
Age (months)				0.75^2^			0.12^2^
Median	40.0	35.0	40.0		45.0	33.0	
Q1, Q3	31.5, 45.0	32.0, 45.0	31.0, 40.0		44.0, 45.0	28.0, 36.0	

Quantitative variables are presented as median, Q1 and Q3. Categorical variables are presented as counts and percentages.

1 Fisher’s Exact Test.

2 Wilcoxon Rank Sum Test.

**Figure 1:**
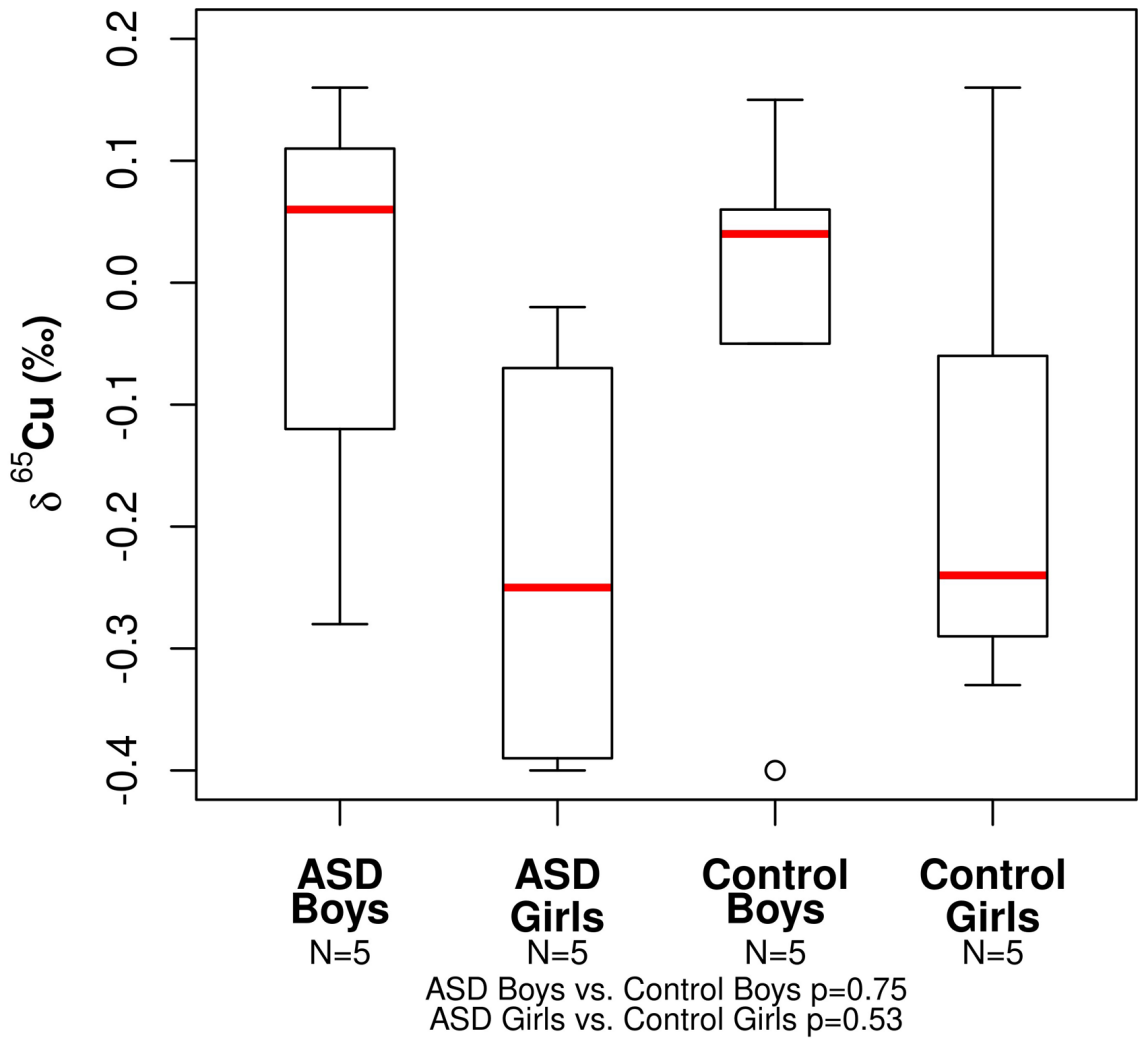
Boxplot for the Cu isotopic composition expressed as *δ*^65^Cu (‰) of bulk serum for the ASD and control populations.

**Figure 2:**
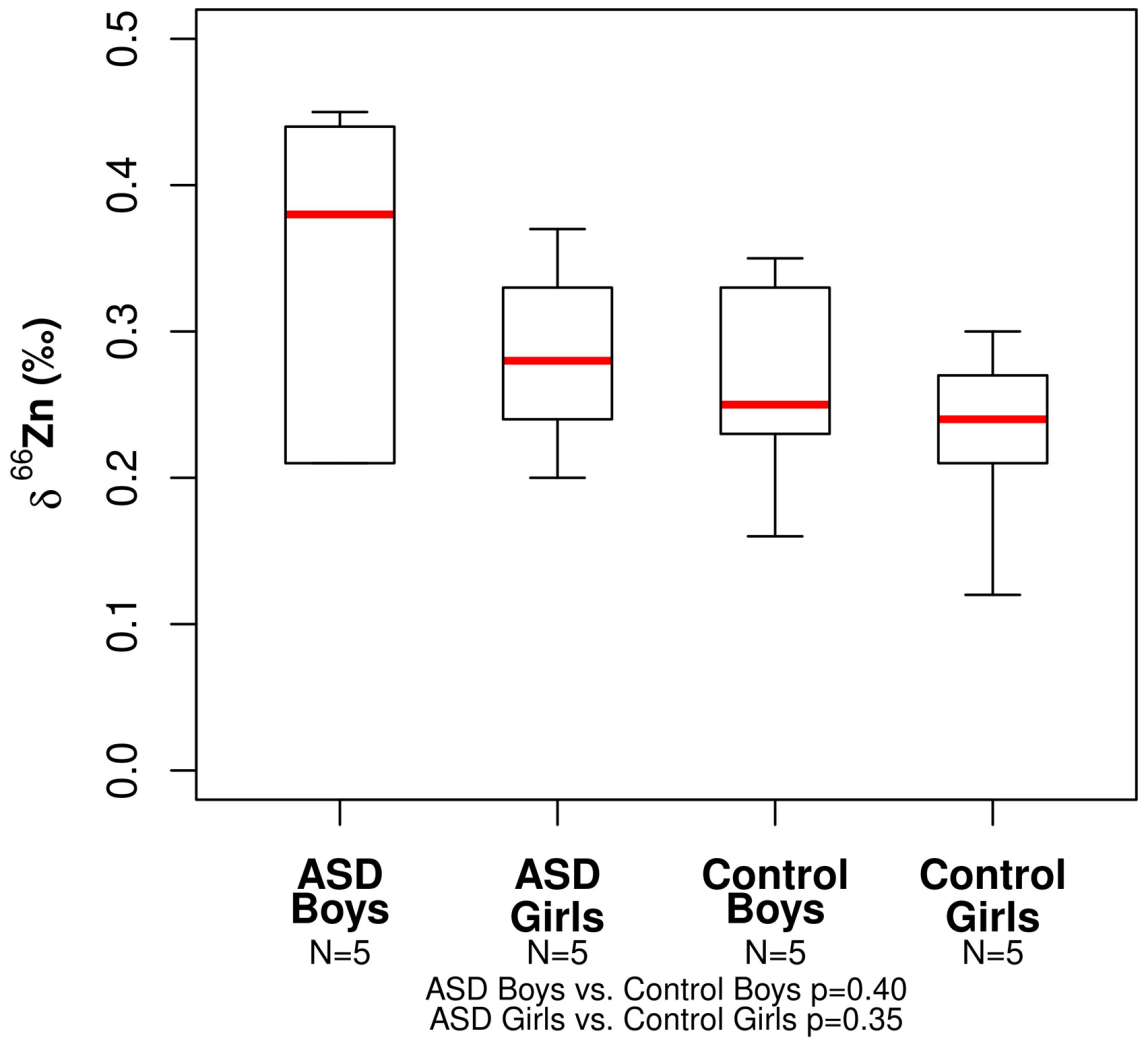
Boxplot for the Zn isotopic composition expressed as *δ*^66^Zn (‰) of bulk serum for the ASD and control populations.

The range in copper *δ*^65^Cu values for all participants was −0.40 to +0.16‰ (Table 2). The mean *δ*^65^Cu value of the serum in both ASD girls (mean = −0.23‰) and control girls (mean = −0.15‰) was relatively depleted in the heavier isotopes compared to both ASD and boy controls (mean = −0.01‰ and −0.04‰, respectively). No statistically significant differences were observed between ASD boys and control boys (*p*− = 0.75) or between ASD girls and control girls (*p* = 0.53; Figure 1). The range in zinc *δ*^66^Zn values for all patients was +0.12 to +0.45‰ (Table 2). No statistically significant differences were observed between the ASD boys and control boys (*p*- = 0.40) or between ASD girls and control girls (*p*- = 0.35). Notably, the spread in measured *δ*^66^Zn values for ASD boys was larger than for the other groups (Figure 2).

**Table 2:** Total Serum Copper and Zinc and Copper *δ*^65^ and Zinc *δ*^64^ results for controls and children with ASD.

Study population	Serum total Cu (mcg/ml)	*δ*^65^Cu (‰)	Serum total Zn (mcg/ml)	*δ*^66^Zn (‰)
ASD boys				
1	1.82	0.11	0.64	0.45
2	1.41	−0.12	0.67	0.44
3	1.74	−0.28	0.86	0.21
4	1.03	0.16	1.07	0.21
5	0.95	0.06	0.66	0.38
Mean ± SD	1.39 ± 0.40	−0.01 ± 0.18	0.78 ± 0.19	0.34 ± 0.12
Control boys				
1	1.14	0.15	0.73	0.33
2	1.31	0.06	1.98	0.23
3	1.45	−0.40	0.80	0.25
4	1.49	0.04	0.53	0.35
5	1.45	−0.05	0.82	0.16
Mean ± SD	1.36 ± 0.14	−0.04 ± 0.21	0.97 ± 0.57	0.26 ± 0.08
ASD girls				
1	1.13	−0.40	0.66	0.20
2	1.19	−0.02	0.59	0.37
3	1.22	−0.39	0.80	0.28
4	1.15	−0.07	0.66	0.33
5	1.05	−0.25	0.68	0.24
Mean ± SD	1.14 ± 0.06	−0.23 ± 0.18	0.68 ± 0.07	0.28 ± 0.07
Control girls				
1	1.19	0.16	0.61	0.21
2	1.18	−0.33	0.64	0.24
3	1.17	−0.24	0.82	0.27
4	1.15	−0.06	0.65	0.12
5	1.22	−0.29	0.77	0.30
Mean ± SD	1.18 ± 0.03	−0.15 ± 0.20	0.70 ± 0.09	0.23 ± 0.07

**Figure 3:**
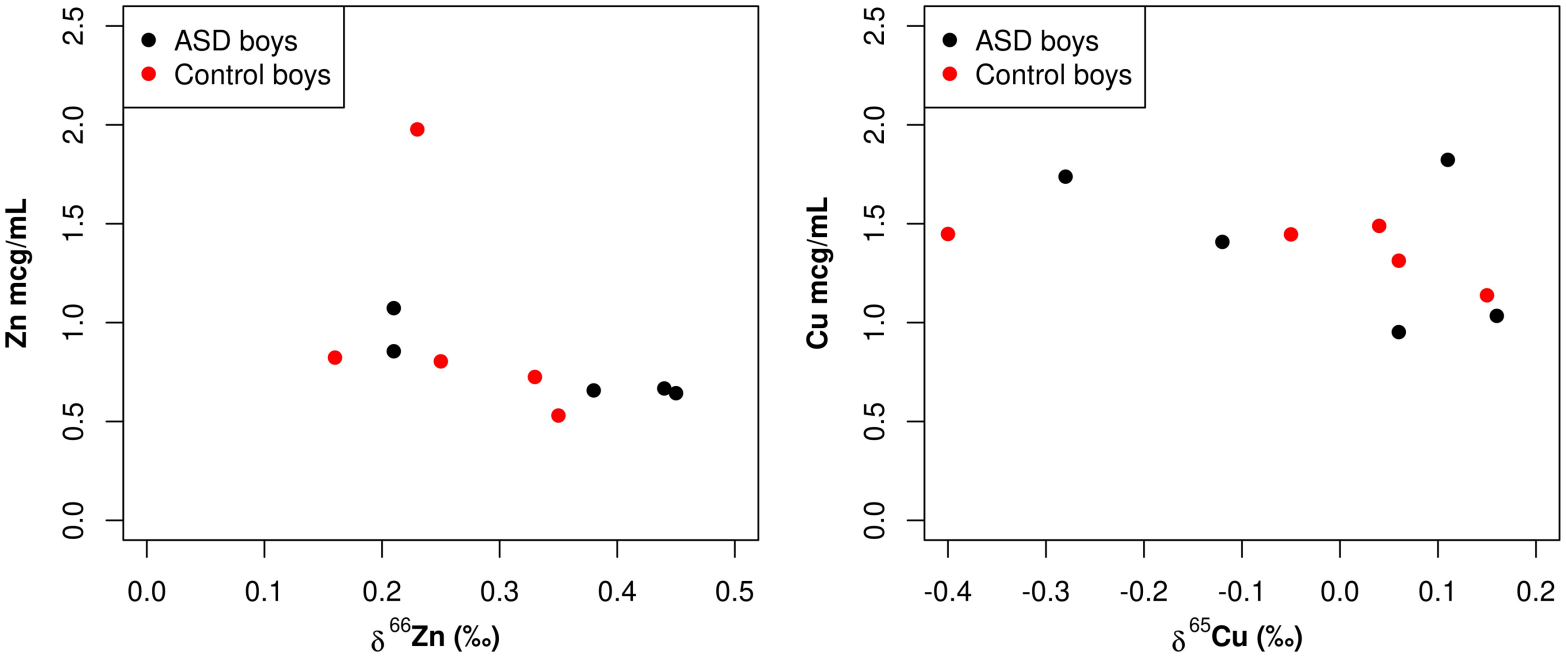
Scatterplots comparing the total element concentration of Cu and Zn (mcg/mL) and the isotopic composition *δ*^65^Cu (‰) and *δ*^66^Zn (‰) of bulk serum in boys.

**Figure 4:**
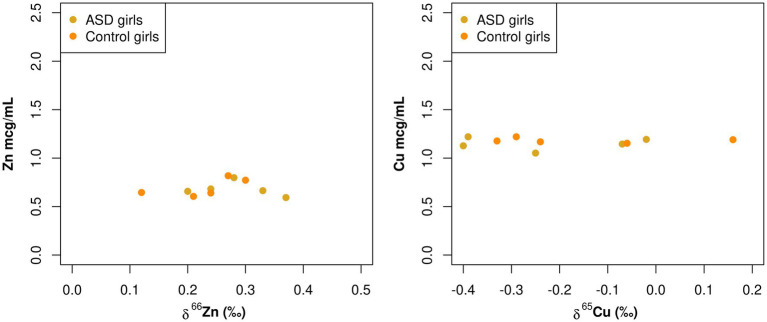
Scatterplots comparing the total element concentration of Cu and Zn (mcg/mL) and the isotopic composition *δ*^65^Cu (‰) and *δ*^66^Zn (‰) of bulk serum in girls.

A statistically significant negative correlation between total Zn serum concentration and Zn isotopic composition was observed in boy patients (−0.58, *p* = 0.020; Table 3; Figure 3). Additionally, while not statistically significant, there was also a negative correlation between total Cu serum concentrations and Cu isotopic composition observed in boy patients (−0.36, *p* = 0.15; Table 3; Figure 3). These same correlations were not apparent in the female population (Zn 0.09, *p* = 0.72, Cu 0.04, *p* = 0.86; Table 3; Figure 4).

**Table 3:** Correlation between total Cu and Zn concentrations and Copper *δ*^65^ and Zinc *δ*^64^ results for controls and children with ASD using the Kendall Tau-b correlation test.

Zn			Cu		
	Kendall Tau-b correlation coefficient	*p*-value		Kendall Tau-b correlation coefficient	*p*-value
Males					
Overall	−0.58	0.020	Overall	−0.36	0.15
ASD	−0.74	0.08	ASD	−0.20	0.62
Control	−0.80	0.050	Control	−0.60	0.14
Females					
Overall	0.09	0.72	Overall	0.04	0.86
ASD	−0.20	0.62	ASD	0.20	0.62
Control	0.40	0.33	Control	−0.20	0.62

**Figure 5:**
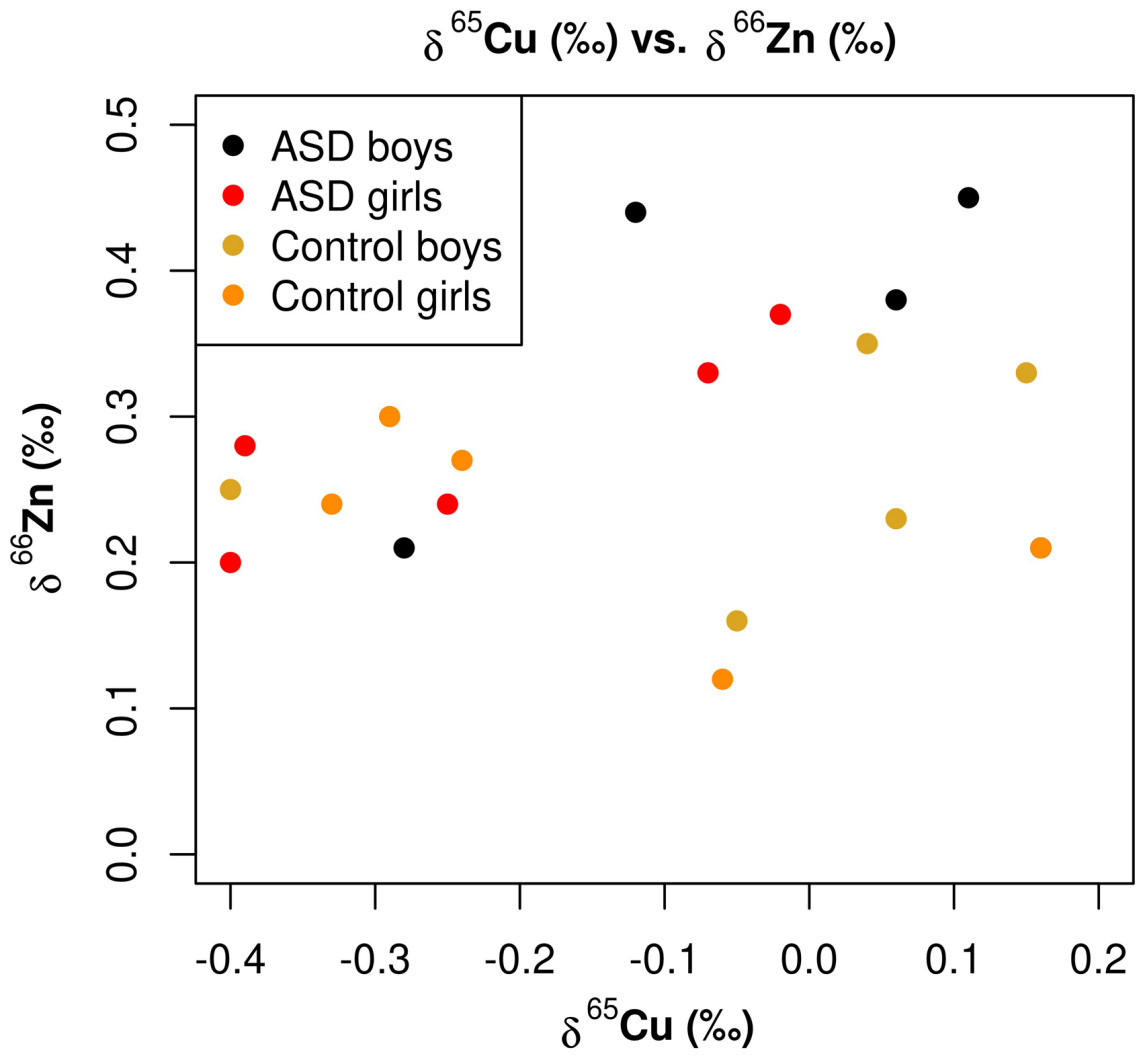
Scatterplot for the *δ*^65^Cu (‰) of bulk serum and the *δ*^66^Zn (‰) of bulk serum for the ASD and control populations.

As depicted in Figure 5, when the copper isotopic composition of the serum was below −0.2‰, as has been observed in multiple studies of healthy adults (Table 4), the *δ*^66^Zn values fell within a narrow range of +0.2 to +0.3‰. However, as the serum became enriched in ^65^Cu, much more variability was seen in Zn isotopic composition. This variation in *δ*^66^Zn values did not appear to be connected to ASD-status, however, and disproportionately affected both the control boys and the ASD boys in this study. It is important to note that in Figure 5, that one boy with ASD had copper and zinc isotope composition values the same as a control girl [*δ*^65^Cu (‰) = 0.16, *δ*^66^Zn (‰) 0.21], respectively, and is represented as only one orange dot on the figure.

**Table 4:** A survey of the copper and zinc isotopic composition of healthy adults in various geographical regions.

Geographical location	Mean age (Years)	Sex	*δ*^65^Cu (‰)	*δ*^66^Zn (‰)	Reference
Belgium	43.8	F (*N* = 10)	−0.29 ± 0.14	0.06 ± 0.13	Hastuti et al. (2020a)
UK	Not specified	M (*N* = 2) F (*N* = 3)	−0.51 ± 0.07*	0.17 ± 0.09	Larner et al. (2015)
France	18–38	F (*N* = 28)	−0.24 ± 0.18	−0.11 ± 0.14^#^	Albarède et al. (2011)
France	18–38	M (*N* = 21)	−0.28 ± 0.20	−0.13 ± 0.05^#^	Albarède et al. (2011)
Japan	56 ± 12	F (*N* = 24)	−0.23 ± 0.13	0.25 ± 0.06	Hastuti et al. (2020b)
Japan	61 ± 11	M (*N* = 23)	−0.20 ± 0.20	0.24 ± 0.10	Hastuti et al. (2020a)
China	27 ± 5	F (*N* = 30)	−0.15 ± 0.17	N/A	Wang et al. (2022)
Belgium	Not specified	M (*N* = 7)	−0.16 ± 0.13	N/A	Lauwens et al. (2018)

Results are presented as Mean +/− 1SD.

* Only females were included in this group.

# The results were adjusted to be on the IRMM3702 scale using the correction factor outlines in Moeller et al. (2012).

## Discussion

To our knowledge this is one of the first studies to measure copper and zinc isotopic fractionation in the serum of both children (25–46 months old) with and without autism spectrum disorder. While there has been a study that measured the *δ*^65^Cu in children with Wilson’s Disease and those without Wilson’s Disease, *δ*^66^Zn values were not measured (Lamboux et al., 2020). Additionally, it is unclear if these samples were age and gender matched as was the case in our study. While we did not find any significant difference in isotopic composition of serum zinc or copper with respect to healthy controls and ASD children, when comparing the isotopic composition of the bulk serum in children compared to adults, the copper isotopic composition of both control girls and ASD girls were closer to the values observed in adults (Table 4). The control and ASD boys had serum that was enriched in the heavier isotopes compared to adult males (Table 4). This appears to be the opposite of what was detected in the study by Lamboux et al. where adult males had serum that was enriched in the heavier isotope when compared to boys (Lamboux et al., 2020). However, like in the study by Lamboux, control boys had much less variability in *δ*^65^Cu values than boys with disease (Lamboux et al., 2020). The zinc isotopic composition of both boys and girls were also enriched in the heavier isotopes than adult counterparts, except for healthy adults from Japan (Table 4).

Furthermore, we observed a negative correlation between total zinc serum concentrations and zjnc isotopic composition in boys and not in girls. Additionally, while not statistically significant, there appears to be a similar association between total copper serum concentrations and copper isotopic composition in boys as well. These finding may indicate that isotopic composition values in addition to total element concentrations for biologically important elements such as copper, zinc and other elements such as selenium and iron may aid in the further study and monitoring of diseases that involve metals dyshomeostasis. It is especially interesting that in the study population, the negative association between total zinc serum concentrations and zinc isotopic composition was only present in boys and not girls. As autism is a disease that significantly effects more boys rather than girls, this association should be further studied. One protein of interest in ASD children is metallothionien, a cysteine rich protein that plays a role in metal detoxification and prevention of oxidative stress. This protein has been shown to be elevated in children with ASD (Vergani et al., 2011) and cysteine would theoretically preferentially bind the lighter isotope of both copper and zinc (Fujii et al., 2013; Tennant et al., 2017). While the negative correlation between Zn concentration and Zn isotopic composition was not associated with ASD-status, but rather appears to be sex-dependent, one possible mechanism to explain this correlation could be an increase in metallothionien expression in those children with elevated Zn concentrations (and Cu). However, without data pertaining to protein expression levels, it is not possible to isolate the specific mechanism for this effect. The mechanism for the poorly controlled zinc isotopic composition observed when serum is enriched in ^65^Cu is still unknown. While this shift in isotopic composition was not necessarily associated with ASD-status, it was observed more often in boys than girls. Copper and zinc-binding proteins such as superoxide-dismutase and metallothionein play a role in the prevention of oxidative stress within the body (Uriu-Adams and Keen, 2005). The high *δ*^65^Cu values in serum combined with variable *δ*^66^Zn values may indicate that these children have higher levels of oxidative stress in the body. This however would need to be further studied using various oxidative stress biomarkers, such as lipid peroxidation, protein oxidation, and elevated antioxidant enzymes could be used to confirm this.

The bulk of the copper in serum is bound to ceruloplasmin, therefore it would be expected that the isotopic composition of the Cu bound to this protein would be reflected in the bulk serum. Lauwens et al. measured copper isotopes in exchangeable and ultrafiltrable portions of serum, with these portions contain multiple proteins including ceruloplasmin, albumin and other low molecular weight proteins (Lauwens et al., 2018). It was observed that these fractions had an isotopic composition very similar to the bulk serum. Therefore, isolating individual proteins in the bulk serum, for example ceruloplasmin, could provide additional insights into the cause of the high *δ*^65^Cu values found in a subset of these children. This could indicate a difference in the copper-loading of this protein in the liver which may not be detrimental to the overall health of the individual but can be detected in isotopic composition due to the sensitivity of this technique to changes in metal processing.

While the sample size of children in this study is small, it indicates that copper and zinc isotopic compositions in children may vary from those values seen in adults. This could indicate differences in metal uptake, incorporation into the body, and subsequent processing in small children compared to adults. While not related to children, research by Jaouen et al., found that copper isotopes composition in blood of postmenopausal women differed from pre-menopausal women and more closely resembled the copper isotopes composition of men (Jaouen and Balter, 2014). Age-related and sex-related differences are sometimes seen in the analysis of multiple trace elements in blood, serum or plasma. For example, Liu et al. found that iron, copper, calcium, and zinc concentrations were strongly correlated with age in a study population of children aged 0–14 (Liu et al., 2012). Furthermore, adult women have higher plasma copper concentration as compared to men, especially women taking oral contraceptives (Milne and Johnson, 1993). If isotopic composition of serum is to be used in the future as an indication of metal dyshomeostasis, especially in children, robust reference range studies that take into consideration age and sex differences must be conducted.

While this study used age matched and sex-matched controls to study copper and zinc isotopic composition of serum in children with autism spectrum disorder, it was not without limitations. As this was an exploratory study, the sample number for this study was small, only containing five individual patients per study group.

## Conclusion

This study provides the first survey of copper and zinc isotopic composition in children with ASD. We did not find any significant difference in isotopic composition of bulk serum zinc or copper with respect to healthy controls and ASD children. We have however shown that copper isotopic composition of serum in boys is enriched in ^65^Cu compared to healthy adults. In both boys and girls, the average isotopic composition of zinc in serum is heavier than healthy adults. Additionally, in boys, there is a negative association between total zinc serum concentrations and zinc isotopic composition. We also observed that in children where copper isotopic composition is enriched in the heavier isotope, that zinc isotopic composition shows more variability. However, in order to determine if these observations may be clinically important, robust serum isotopic composition reference range studies that are age and sex dependent must be conducted. This is necessary before isotopic composition analysis can be used as a potential clinical tool for monitoring patients over time who have disorders affecting trace metals homeostasis.

## Data availability statement

The original contributions presented in the study are included in the article/supplementary material, further inquiries can be directed to the corresponding author/s.

## Ethics statement

The studies involving human participants were reviewed and approved by Mayo Clinic Institutional Review Board. Written informed consent to participate in this study was provided by the participants’ legal guardian/next of kin.

## Author contributions

All authors listed have made a substantial, direct, and intellectual contribution to the work and approved it for publication.

## Funding

MEW laboratory is supported through a Discovery Grant from the Natural Sciences and Engineering Research Council of Canada (NSERC). MP thanks the Department of Radiology, Mayo Clinic, Rochester, MN for his time to work on this study. A portion of this study was funded by the Children’s Research Center, Mayo Clinic, Rochester, MN.

## Conflict of interest

The authors declare that the research was conducted in the absence of any commercial or financial relationships that could be construed as a potential conflict of interest.

## Publisher’s note

All claims expressed in this article are solely those of the authors and do not necessarily represent those of their affiliated organizations, or those of the publisher, the editors and the reviewers. Any product that may be evaluated in this article, or claim that may be made by its manufacturer, is not guaranteed or endorsed by the publisher.
